# Intestinal Microbiome of Newly Diagnosed Patients With Neovascular Age-Related Macular Degeneration: A 16S rRNA Gene Sequencing Study

**DOI:** 10.7759/cureus.103984

**Published:** 2026-02-20

**Authors:** Andreas Katsanos, Konstantinos Benekos, Maria Karavasili, Konstantina Gorgoli, Charilaos Kostoulas, Konstantina Gartzonika, Dimitrios K Christodoulou, Konstantinos Katsanos, Ioannis Georgiou

**Affiliations:** 1 Department of Ophthalmology, University Hospital of Ioannina, Ioannina, GRC; 2 Laboratory of Medical Genetics, Faculty of Medicine, School of Health Sciences, University of Ioannina, Ioannina, GRC; 3 Department of Microbiology, University Hospital of Ioannina, Ioannina, GRC; 4 Department of Gastroenterology and Hepatology, University Hospital of Ioannina, Ioannina, GRC

**Keywords:** age-related macular degeneration (amd), amd, gut microbiome, intestinal microbiome, next generation sequencing (ngs)

## Abstract

Background: This study aims to explore differences in the intestinal microbiome between patients with newly diagnosed neovascular age-related macular degeneration (AMD) and controls using 16S rRNA gene sequencing.

Methodology: In this cross-sectional study, stool samples from newly diagnosed White patients with neovascular AMD and controls were used for the assessment of the intestinal microbiome. The DNeasy PowerSoil Pro Kit (QIAGEN, Hilden, Germany) was used to extract microbial DNA before sequencing the V3-V4 hypervariable region of the 16S rRNA gene on the Illumina MiSeq system (Illumina, San Diego, CA). Bioinformatic analysis was performed on the Nephele platform using the DADA2 pipeline in R (ClinicalTrials.gov identifier: NCT05757674).

Results: Thirty-three patients (age: 75 ± 7 years, 17 women) and 34 age- and sex-matched controls (age: 73 ± 7 years, 18 women) were analyzed. No differences in height, weight, body mass index, smoking, or systemic comorbidities were noted between the groups. The most prevalent phyla in both groups were Firmicutes, Bacteroidota, Proteobacteria, and Actinobacteria. The most prevalent genus was Bacteroides in both groups. Neither alpha nor beta diversity was different among the groups. The differential abundance analysis using Analysis of Compositions of Microbiomes with Bias Correction 2 (ANCOM-BC2) showed that some Amplicon Sequence Variants (ASVs) from the Coprococcus genus were more abundant in controls than in patients with AMD, whereas several ASVs from Bacteroides were more abundant in the AMD group.

Conclusions: In our sample, the intestinal microbiome of newly diagnosed patients with neovascular age-related AMD showed some small but noteworthy differences compared to matched healthy controls. Some Bacteroides ASVs were enriched in AMD patients, while certain Coprococcus ASVs were more abundant in controls.

## Introduction

Age-related macular degeneration (AMD) is the leading cause of irreversible blindness in individuals aged 60 and older in the developed world [1]. It is estimated that AMD affects approximately 200 million people worldwide, a figure which is expected to rise to 300 million by 2040, largely due to population aging [2].

AMD is a complex eye disorder influenced by diverse risk factors, such as age, genetics, environment, and the immune system [3]. The interaction of the predisposing risk factors that ultimately lead to the disease remains unknown. The primary symptom of AMD is a deterioration of central vision, as the condition primarily affects the macula, i.e., the anatomical area responsible for the central vision [4]. Depending on the nature of the lesion, the disease can be divided into two different clinical entities: neovascular or exudative (often called *wet*), and nonexudative (often called *dry*) AMD. The neovascular type is the one most commonly leading to significant visual impairment [5].

It has been suggested that the eye may be affected by the microbial profile of the intestine, both in animal models and in humans [6]. Purportedly, alterations in the constitution and metabolism of intestinal microbes may create an unfavorable environment (*dysbiosis*) that causes homeostatic imbalance and disease not only inside the gut but also in distant organs. This hypothetical relationship between the gut and the eye is referred to as the *gut-eye axis* and has been linked to ocular disorders such as keratitis, uveitis, or glaucoma [6,7]. In addition, gut microbiome dysbiosis has been hypothesized to be associated with the development and progression of AMD in humans [8], and thus, the relationship between intestinal dysbiosis and the onset of AMD is currently a matter of great interest.

Published studies characterizing the gut microbiome profile of patients with AMD are currently scarce, and their findings are inconsistent [4,9,10]. The primary goal of this study was to explore any differences in the gut microbiome of white patients with newly diagnosed AMD and, secondly, to identify potential microbial biomarkers of this vision-threatening disorder using 16S rRNA gene sequencing.

## Materials and methods

The study design was assessed and approved by the local ethics committee of the University Hospital of Ioannina (protocol number: 141/14-2-2020). The research adhered to the principles outlined in the Declaration of Helsinki. This project is registered with ClinicalTrials.gov (NCT05757674). Before enrollment in the study, each participant signed a fully informed consent form.

In this study, only participants who met the following criteria were included. Patients with newly diagnosed AMD who were referred to or followed at the Ophthalmology Clinic of the University Hospital of Ioannina or the General Hospital of Ioannina were eligible. Healthy controls without signs of AMD were recruited from the Ophthalmology Clinic of the University Hospital of Ioannina and underwent a comprehensive ocular examination, including visual acuity assessment, dilated fundoscopy, and optical coherence tomography (OCT). All participants were required to be at least 60 years of age and capable of providing written informed consent and understanding the requirements of the study.

Individuals who had previously received any type of treatment for wet AMD were excluded. Additionally, participants who had received antibiotics or probiotics within one month before their inclusion in the study were also excluded.

First-morning fecal samples were self-collected using a disposable sterile plastic wrap. Each participant collected stool from a single bowel movement and transferred the sample into a sterile container using the spoon provided as part of the collection kit. After the sampling, the fecal samples were immediately frozen to -20 °C before DNA extraction. The DNeasy PowerSoil Pro kit (QIAGEN, Hilden, Germany) was used to extract microbial DNA from the fecal samples according to the manufacturer’s instructions, and the DNA was stored at -20 °C. The quantity and quality of isolated DNA were assessed using the fluorometer Qubit 3 (Thermo Fisher Scientific, Waltham, MA) and Q3000 UV Spectrophotometer (Quawell Technology, San Jose, CA), respectively. Library construction and sequencing of the V3-V4 hypervariable region of 16S rRNA were performed. Polymerase chain reaction (PCR) amplification of the V3-V4 region was performed using the KAPA HiFi HotStart ReadyMix (Roche Sequencing Solutions, Pleasanton, CA) with primers 341F (GTCTCGTGGGCTCGGAGATGTGTATAAGAGACAG-CCTACGGGNGGCWGCAG) and 805R (GTCTCGTGGGCTCGGAGATGTGTATAAGAGACAG-GACTACHVGGGTATCTAATCC). Illumina adapter overhang nucleotide sequences (bold sequences) were added to both primers [11]. The generated libraries were pooled at equimolar concentrations and quantified by quantitative polymerase chain reaction (qPCR) using the KAPA Library Quantification Assay (Roche Sequencing Solutions) in accordance with the manufacturer’s protocol. The resulting libraries were sequenced on the Illumina MiSeq system using the MiSeq Reagent Kit v2 (Illumina, San Diego, CA).

The paired-end FASTQ files generated after sequencing were analyzed using the DADA2 pipeline [12] on the Nephele platform [13]. This analysis included inspecting the read quality profile, filtering and trimming the reads with a maximum expected error threshold of 2, chimera removal, merging the pair reads, constructing the sequence table, and taxonomic assignment. Both forward and reverse reads were trimmed to 240 bp. Regarding the taxonomy assignment, Silva database version 138.1 was used [14].

After the DADA2 pipeline [12] was completed, the output files were imported into the phyloseq R package (version 1.54.0) [15] to complete the statistical analysis and visualization of the results. Before calculating the diversity metrics, the samples were rarefied to 90% of the lowest sequence depth observed in a sample (except for Aitchison distance calculations [16]).

For non-phylogenetic Alpha diversity analysis, the Shannon and Simpson indices were calculated and compared between the two groups with the Wilcoxon rank-sum test (significant results, *P *< 0.05). 

Regarding the beta diversity, the Bray-Curtis dissimilarity index at the Amplicon Sequence Variant (ASV) level was calculated and used to visualize beta diversity on principal coordinate analysis (PCoA) plots. ASVs can be defined as the distinct DNA sequences recovered from the sequencing process of the 16S rRNA gene of different microbes. Each ASV represents a unique sequence within a sample that can be matched to a specific microbe, typically after rigorous error correction and quality control. PCoA simplifies microbiome datasets by reducing their dimensionality, allowing beta diversity relationships to be visualized in two- or three-dimensional scatterplots. In addition, the difference in beta diversity between the two groups was assessed with PERMANOVA using the adonis function of the vegan package in R, version 2.7.2 [17]. Furthermore, after centered log-ratio transformation (CLR) of the data, Aitchison distance was calculated at the ASV level and plotted in order to validate the previous metrics, as this is the proposed method that accounts for the compositionality nature of the microbiome dataset [16].

Finally, regarding the differential abundance analysis, Analysis of Compositions of Microbiomes with Bias Correction 2 (ANCOM-BC2) [18] was utilized in order to identify taxa that have significantly different abundance between the two tested groups. Data from the phyloseq object were imported into ANCOM-BC2 (version 2.12.0) [18] in R. Taxa presented in less than 20% of the samples were excluded from the analysis. We opted to perform the differential abundance analysis at the lowest taxonomic level of our data. As suggested, in order to reduce the chance of false positives arising from multiple tests between the taxa of the two groups, we opted for the Holm-Bonferroni method [19]. 

## Results

**Figure 1:**
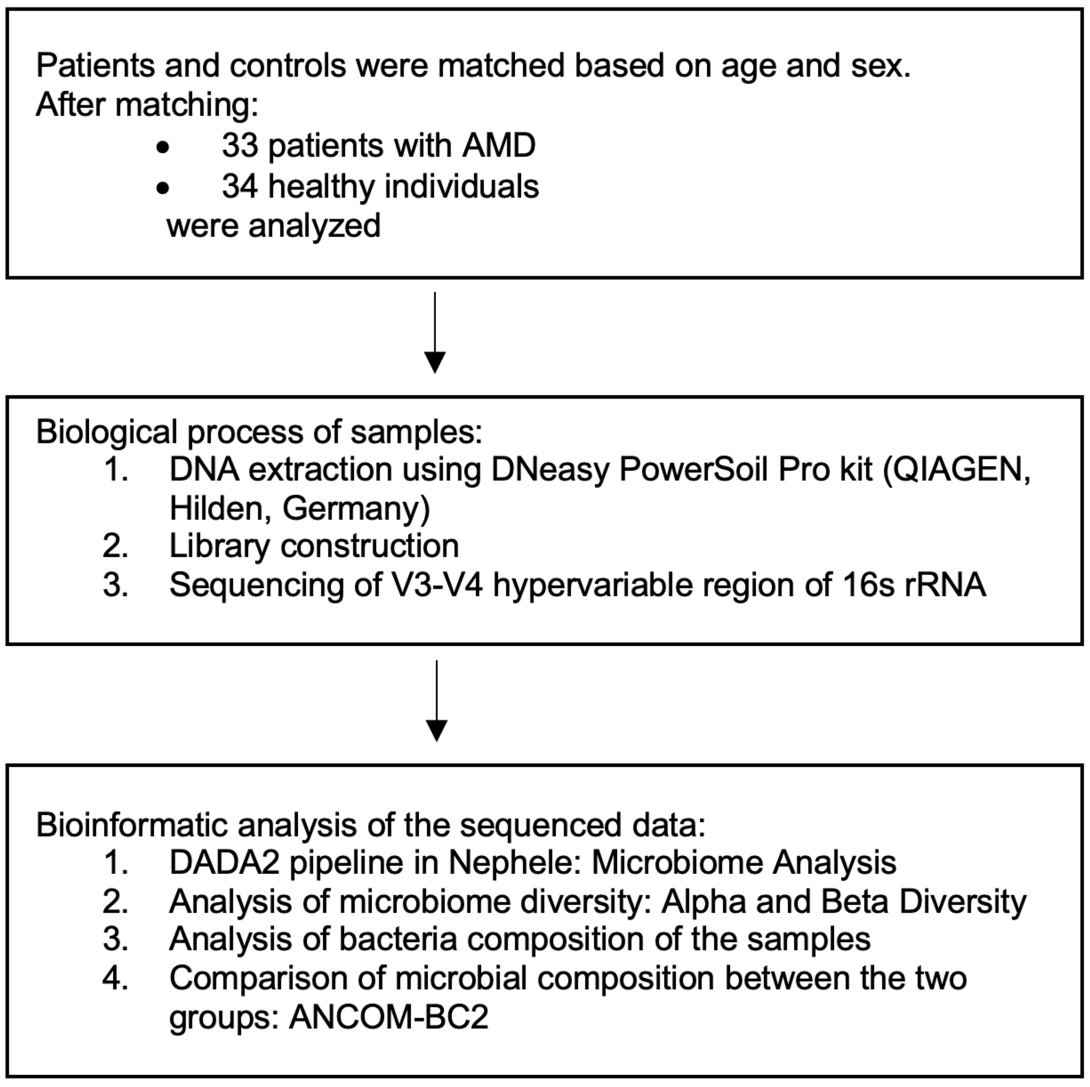
Study flowchart. AMD, age-related macular degeneration; ANCOM-BC2, Analysis of Compositions of Microbiomes with Bias Correction 2

The study flowchart is presented in Figure 1.

All participants of this study were White residents of the Northwestern region of Epirus and Western Macedonia, Greece. Data from 33 newly diagnosed patients with AMD and 34 age- and sex-matched healthy controls were analyzed. Clinical characteristics such as body mass index (BMI), hypertension, hypercholesterolemia, diabetes, and smoking habits were comparable between the two groups. None of the participants were vegan, vegetarian, or followed any particular diet demanding the exclusion or the excessive consumption of a specific food group. The baseline characteristics of the study participants are presented in detail in Table 1.

**Table 1:** Participant characteristics. ^a^Mean (SD), median (Q1 to Q3), *n*/*N* (%) ^b^t-test or Mann-Whitney U test for continuous variables; Fisher’s exact test for categorical variables.

Characteristics	Patients with AMD (*n *= 33)^a^	Controls (*n* = 34)^a^	*P*-value^b^
Age (years)	75 (7)	73 (7)	0.10
Height (m)	1.65 (0.10)	1.66 (0.08)	0.7
Unknown	1	0	
Weight (kg)	75 (69-84)	76 (68-84)	>0.9
Unknown	1	0	
BMI (kg/m²)	28.4 (25.9-31.3)	27.0 (25.9-30.1)	0.5
Unknown	1	0	
Hypertension	18/33 (55%)	19/34 (56%)	>0.9
Hypercholesterolemia	8/33 (24%)	13/34 (38%)	0.2
Smoking	10/33 (30%)	6/34 (18%)	0.2
Sex			>0.9
Women	17/33 (52%)	18/34 (53%)	
Men	16/33 (48%)	16/34 (47%)	
Diabetes type 2	6/33 (18%)	6/34 (18%)	>0.9

**Figure 2:**
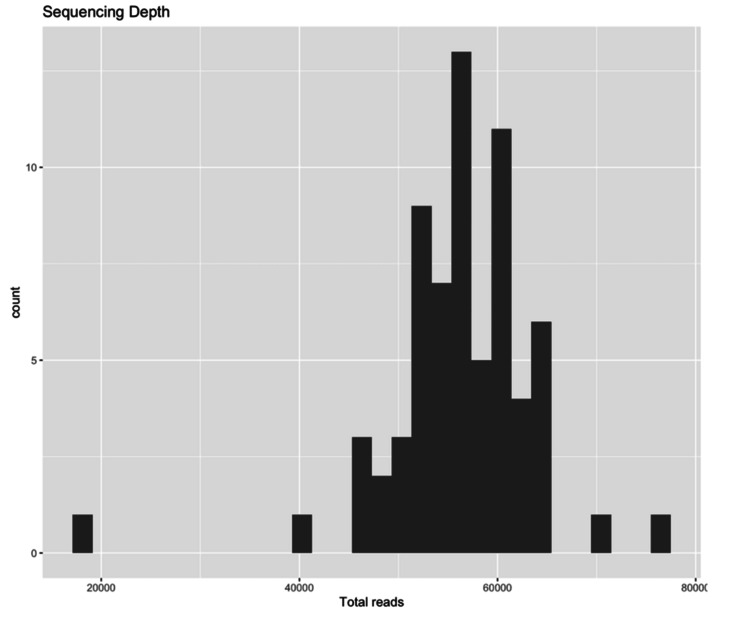
Distribution of sample read counts.

After filtering, trimming, and removing the chimeras using the DADA2 pipeline in Nephele [13], the median sequencing depth across the 67 samples of this study was 56735 reads (interquartile range (IQR): 52672-60643). The distribution of the read numbers after this process is shown in Figure 2.

**Figure 3:**
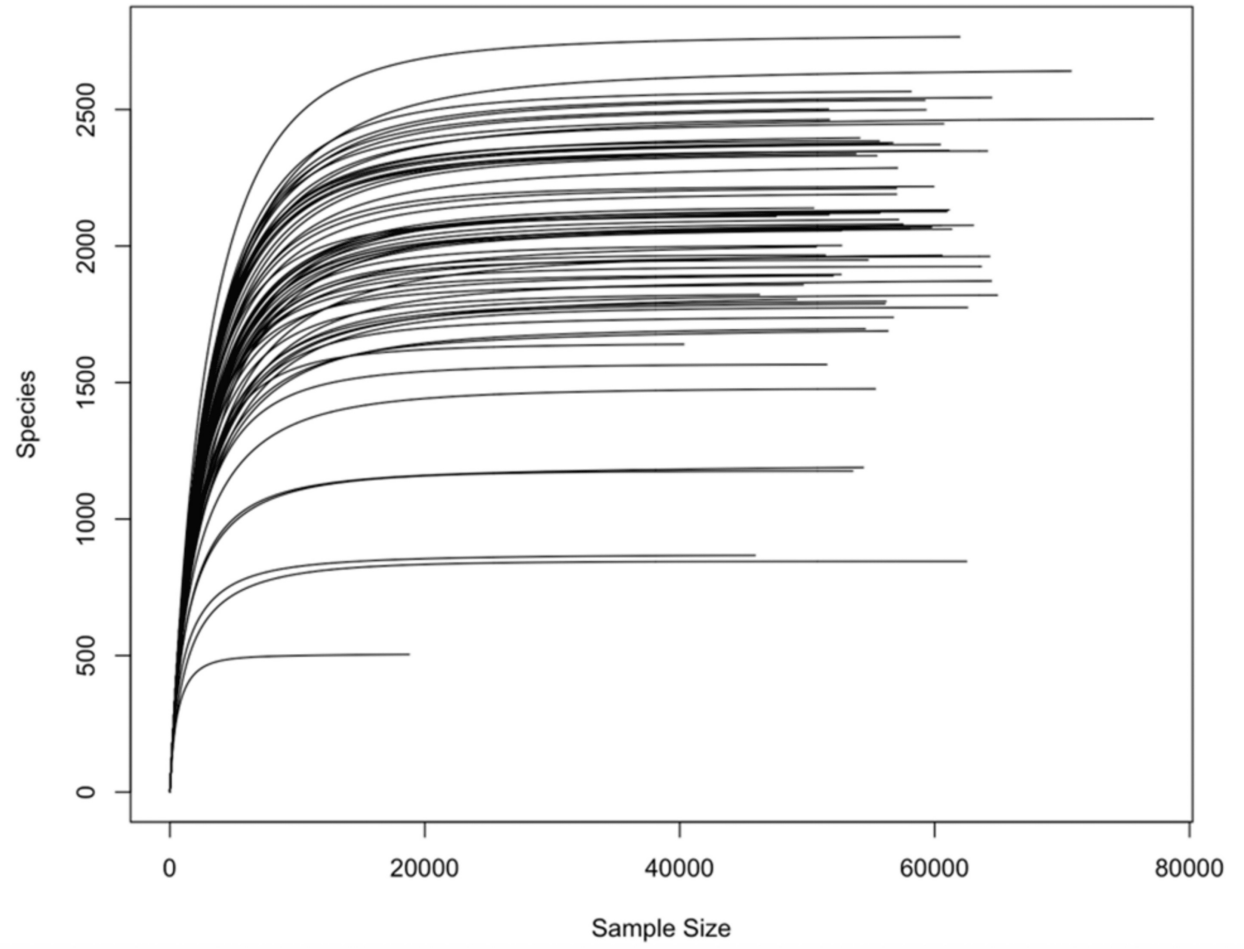
Rarefaction curves.

The rarefaction curves (Figure 3) indicated that as the number of reads expanded, the curves flattened, indicating comprehensive coverage of almost all species by the sequencing depth. These results confirm the sufficiency of the sequencing data, suggesting that acquiring more sequencing data would not reveal additional ASVs or species. The minimum number of reads observed was 18774. To reduce bias caused by varying sequencing depth, all samples were rarefied to a sequence depth of 16896.6 before calculating alpha and beta diversity, except when calculating Aitchison distances, which involves its own normalization process [16].

**Figure 4:**
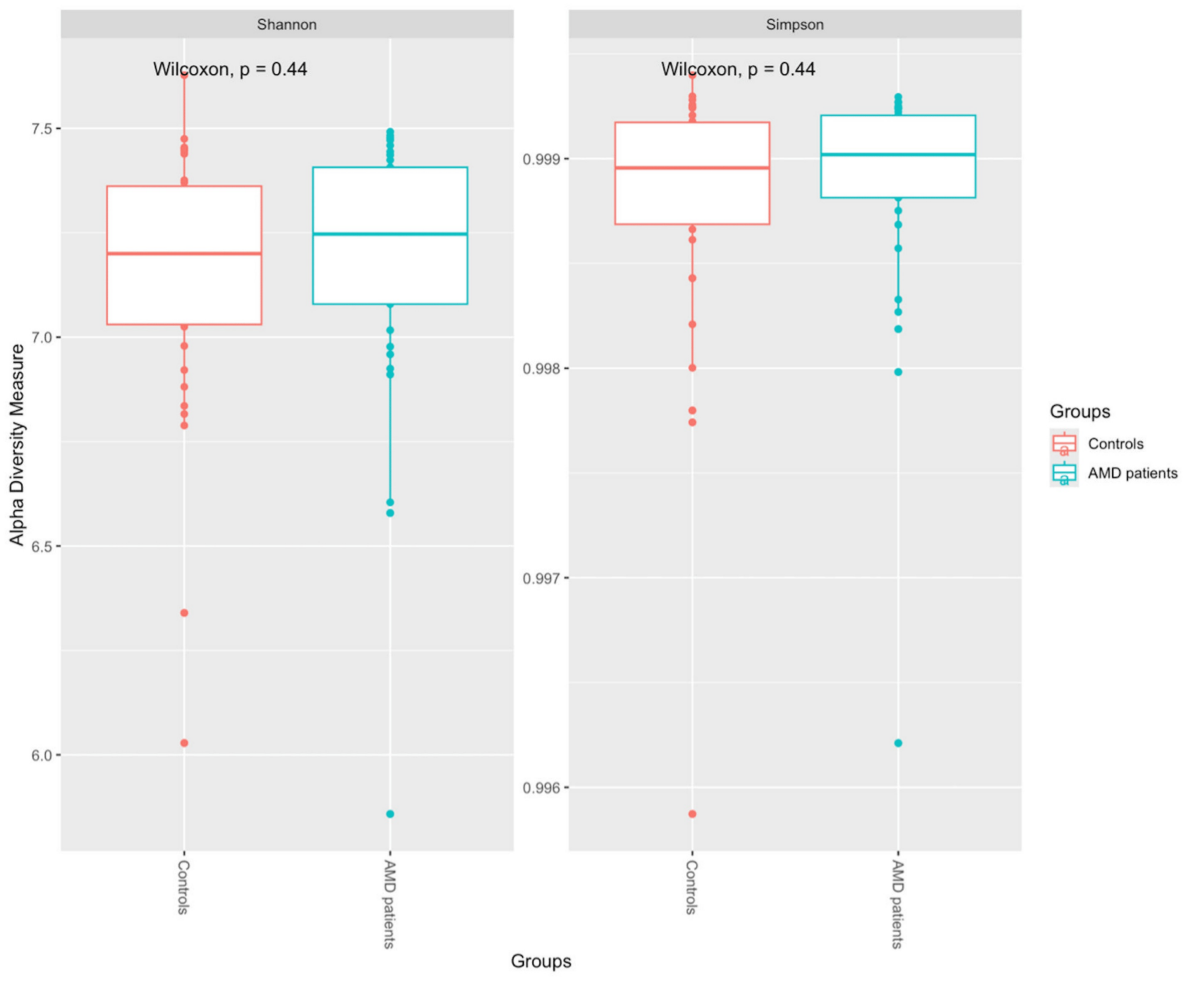
Measures of alpha diversity (Shannon and Simpson diversity indices). AMD, age-related macular degeneration

In the non-phylogenetic alpha diversity analysis (Figure 4), at the ASV level, the Simpson and Shannon indices in the fecal samples of patients with AMD did not differ from those of healthy individuals (*P* = 0.44 for both comparisons).

**Figure 5:**
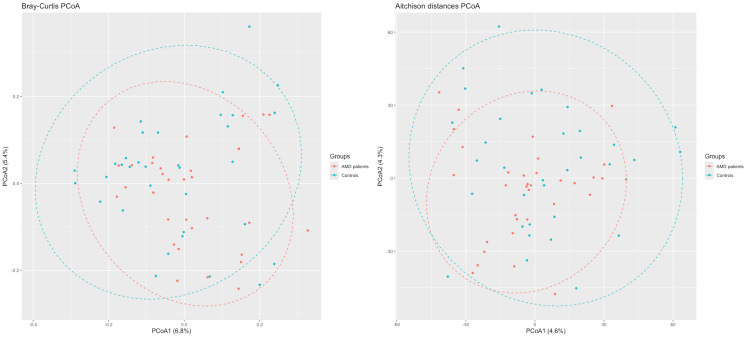
Principal coordinates analysis (PCoA) based on Bray-Curtis and Aitchison distances.

The PERMANOVA significance test for group-level differences revealed no significant differences between the two groups (*P* > 0.05). PCoA plots based on both Bray-Curtis and Aitchison distances are shown in Figure 5.

The composition of intestinal microbiota in the two groups was analyzed at different taxonomic levels. Table 2 presents the number of species identified at each taxonomic level in all samples.

**Table 2:** Number of species identified at each taxonomic level.

Taxonomic levels	Total number of different species identified
Kingdom	1
Phylum	11
Class	17
Order	43
Family	82
Genus	279

**Figure 6:**
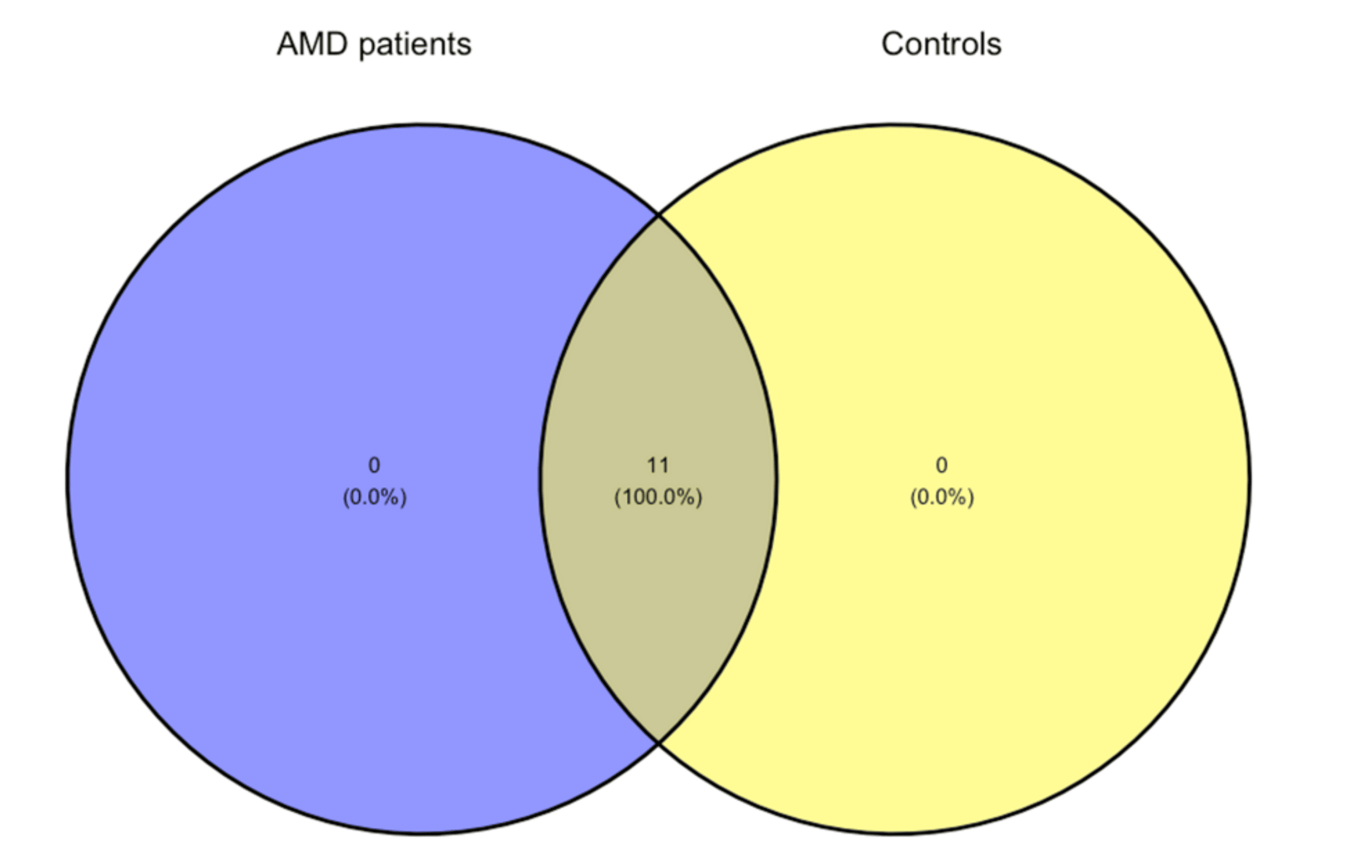
Venn diagram showing the number of shared and unique phyla. AMD, age-related macular degeneration

**Figure 7:**
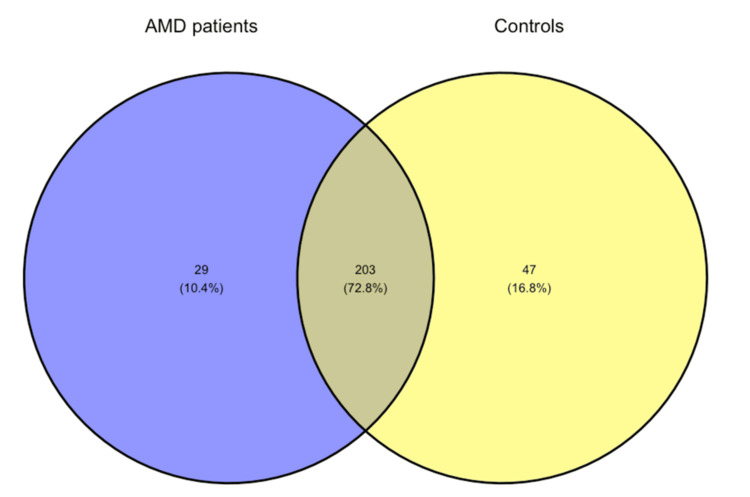
Venn diagram showing the number of shared and unique genera. AMD, age-related macular degeneration

In both groups, 11 phyla were identified. In contrast, at the genus level, 232 genera were identified in the AMD group and 250 in the control group, with only 203 genera overlapping between the two tested groups. The corresponding Venn diagrams can be found in Figures 6-7.

The most prevalent phyla identified in both groups were Firmicutes, Bacteroidota, Proteobacteria, and Actinobacteria, with the first two together comprising around 90% of the total bacterial population in both groups (Table 3).

**Table 3:** Relative abundances at phylum and genus levels. AMD, age-related macular degeneration

	Patients with AMD, Mean relative abundance (% ± SD)	Controls, Mean relative abundance (% ± SD)
Phylum		
Firmicutes	69.7 ± 9.03	70.96 ± 8.71
Bacteroidota	20.61 ± 6.70	19.11 ± 7.52
Actinobacteriota	5.35 ± 5.05	5.11 ± 3.82
Proteobacteria	3.33 ± 6.05	3.82 ± 6.33
Genus		
Bacteroides	15.04 ± 6.74	14.06 ± 8.34
Blautia	10.56 ± 4.83	10.20 ± 6.55
Faecalibacterium	9.5 ± 4.21	8.49 ± 5.54
Agathobacter	3.93 ± 2.99	3.92 ± 3.19
Ruminococcus	3.88 ± 3.20	4.03 ± 4.36
Subdoligranulum	2.61 ± 1.86	3.92 ± 2.76

**Figure 8:**
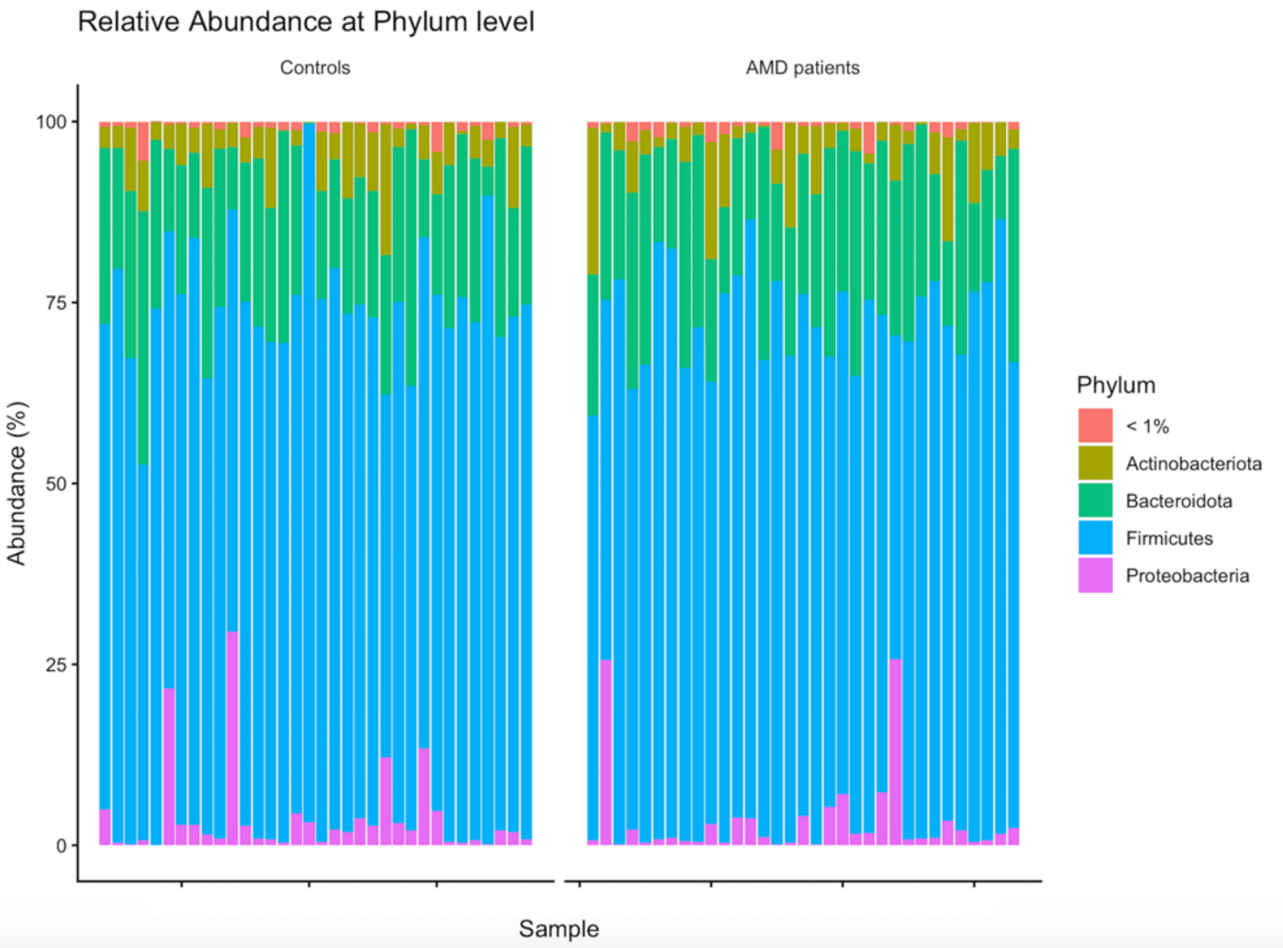
Relative abundance at the phylum level. AMD, age-related macular degeneration

**Figure 9:**
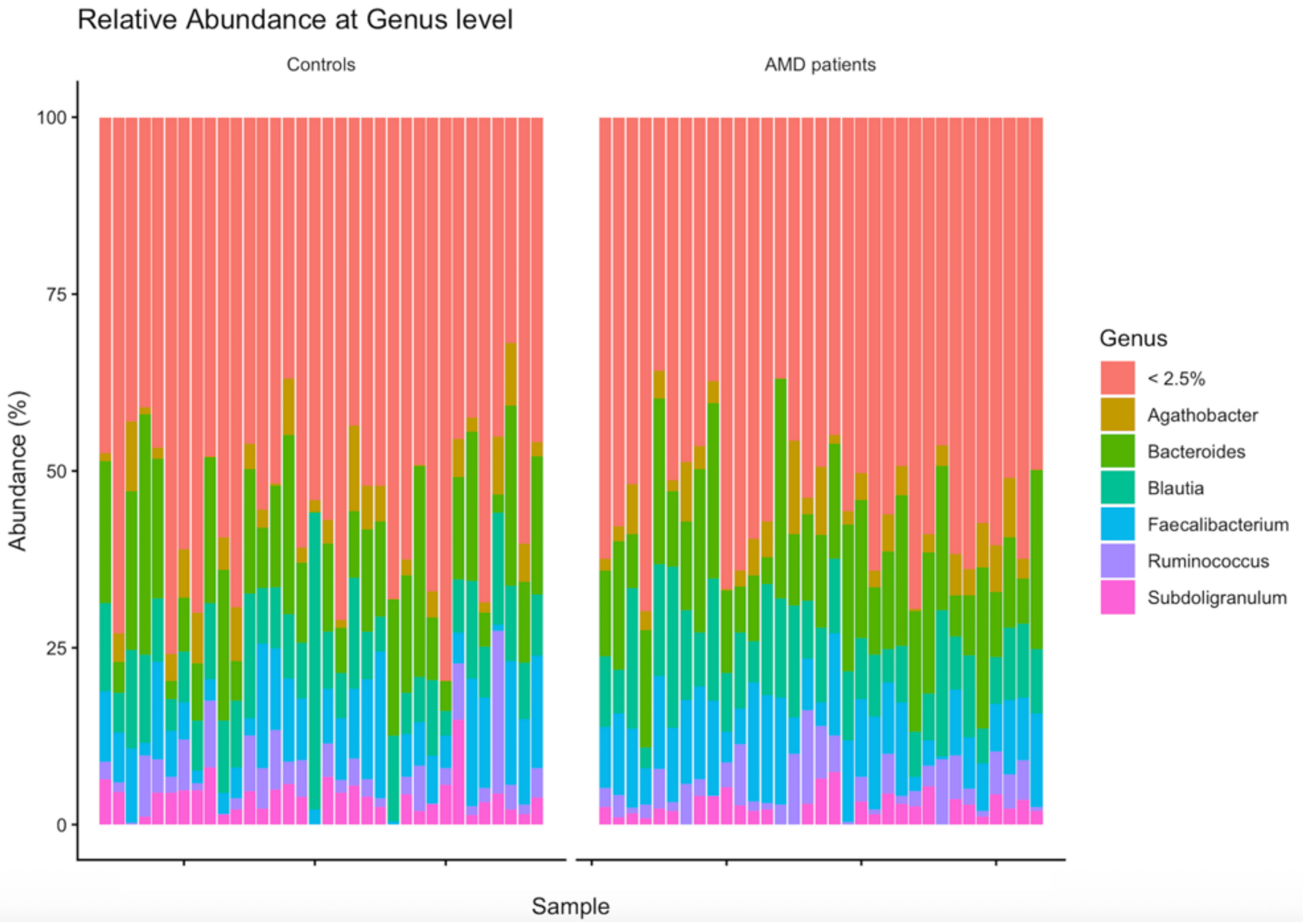
Relative abundance at the genus level. AMD, age-related macular degeneration

At the genus level, Bacteroides was the most abundant in both groups, accounting for around 15% of the total bacterial composition in both groups of this study (Table 3). Bar charts illustrate the relative abundance at both levels (Figures 8-9).

**Figure 10:**
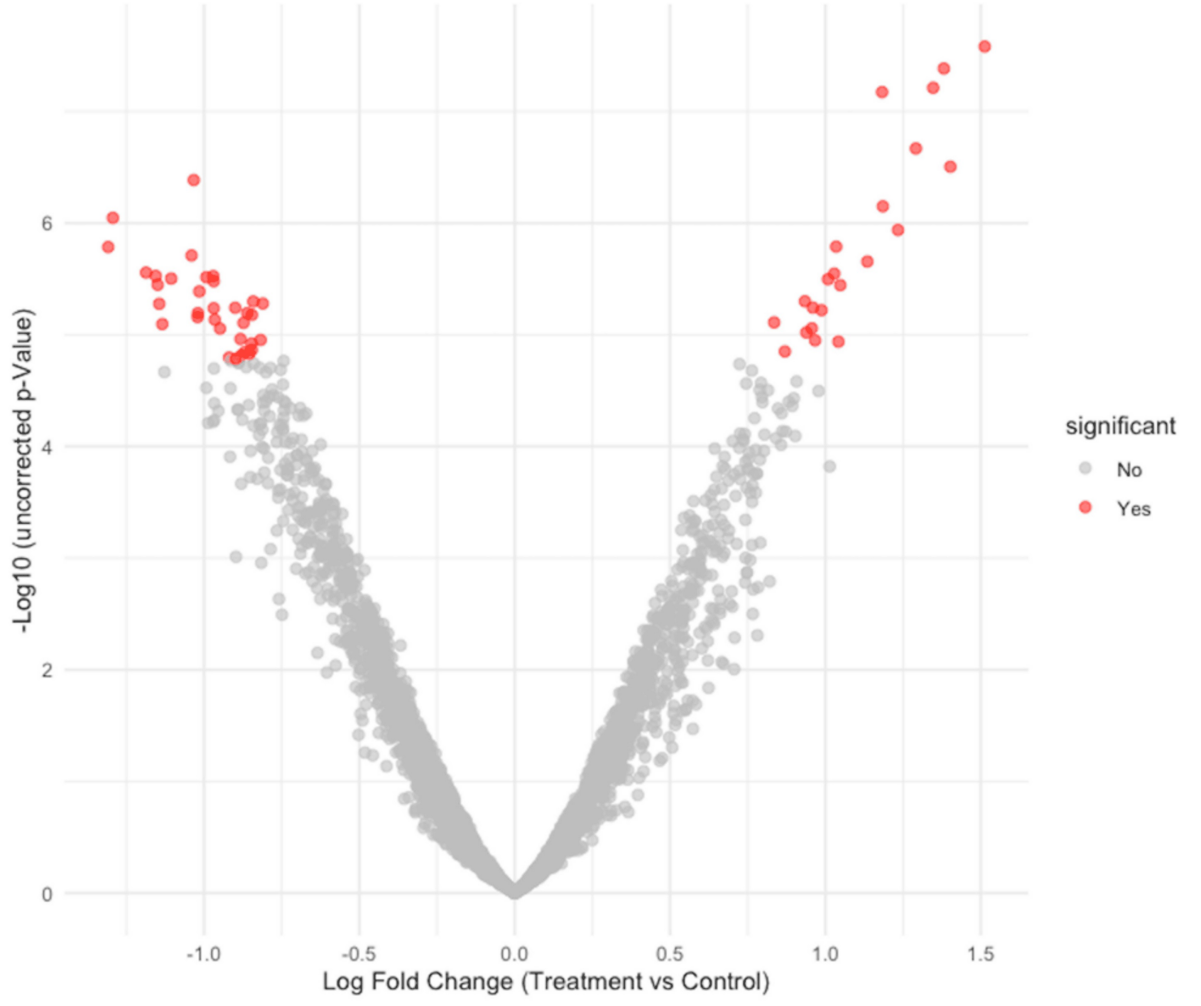
Volcano plot of ANCOM-BC2 results. ANCOM-BC2, Analysis of Compositions of Microbiomes with Bias Correction 2

**Figure 11:**
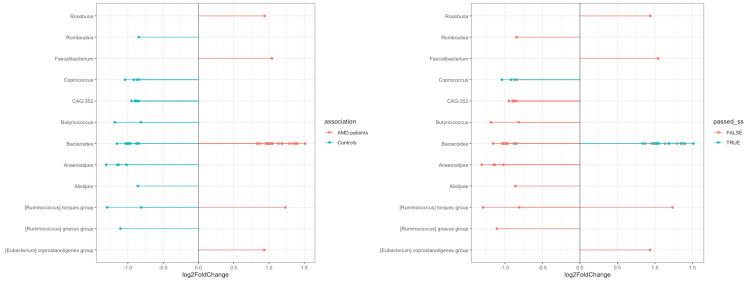
Altered microbiota at the ASV level. ASV, Amplicon Sequence Variant

The differential analysis at the ASV level, having controls as the reference group, showed certain differences between the two compared groups, as presented in the following figures (Figures 10-11). A sensitivity analysis was conducted by the ANCOM-BC2 [18] tool itself to evaluate the impact of applying various pseudo-counts on zero counts for each taxon. Most of the identified taxa did not consistently show significance across the different pseudo-counts, leading to their classification as sensitive to pseudo-counts (passed_ss = FALSE), which, in fact, suggests that they were likely false positives. Despite the sensitivity analysis reducing the certainty of evidence for most results, some differences in the differential abundance analysis remained significant. All identified ASVs from the Coprococcus genus that passed the sensitivity analysis were more abundant in controls than in patients with AMD, whereas most Bacteroides that also passed the analysis were more abundant in the AMD group.

## Discussion

It has been hypothesized that the microorganisms that inhabit the human gut may be the missing link between genetic and environmental factors and the pathogenesis of AMD [8]. Recent studies have suggested the existence of a *gut-retina axis* by detecting changes in the gut microbiome of patients with various ocular diseases [20,21]. The current study has identified only a few differences between patients with AMD and controls. While our findings contrast with some previous studies that reported differences in the relative abundances of even the main gut phyla [4], they are in line with other studies that found more limited microbiome differences between patients with AMD and controls [9,10].

This study analyzed 33 patients who had recently been diagnosed with neovascular AMD and 34 age- and sex-matched healthy controls from a racially and geographically well-defined population. Clinical characteristics such as BMI, arterial hypertension, hypercholesterolemia, diabetes mellitus, and smoking were similar across the two groups so as to limit their confounding effect. Our study revealed some differences in the gut microbiome of newly diagnosed neovascular patients with AMD compared to healthy controls.

Consistent with other gut microbiome studies [22,23], the most frequently occurring bacterial phyla in the stool samples of both groups were Firmicutes, Bacteroidetes, Proteobacteria, and Actinobacteria. In both groups, Firmicutes and Bacteroidetes were the dominant phyla, representing around 90% of the gut microbiota, which is in line with what is typically observed in healthy individuals [22-24]. At the genus level, Agathobacter, Bacteroides, Blautia, Faecalibacterium, Ruminococcus, and Subdoligranulum were the most predominant genera in both groups.

Regarding alpha diversity, i.e., the *within-sample* diversity, which describes the richness and evenness of the microbiome in each sample, neither the Simpson's nor the Shannon index showed any difference between the tested groups. For beta diversity, which quantifies differences in the overall taxonomic composition between two samples, we used both the Bray-Curtis dissimilarity index and the Aitchison distance to account for the compositionality of the microbiome data [16]. Neither of these showed any significant difference between the two groups, which is further supported by the PERMANOVA test, indicating no statistically significant difference between them. 

To test the differential abundance in ASV level between the two groups, the ANCOM-BC2 tool [18] was used. It has been shown that compared to other mainstream methods such as DESeq2 [25] or LefSe [26], the ANCOM-BC can produce the most consistent results, taking into account the compositionality of a microbiome dataset [27]. The newer version (i.e., ANCOM-BC2) [18] expands and improves upon the ANCOM-BC method introduced by Lin and Peddada in 2020 [19] in several important aspects. Firstly, using various methods, ANCOM-BC2 [18] adjusts for multiple comparisons, thus reducing false discovery rates. This correction reduces the likelihood of false positives when testing a large number of hypotheses, for example, when evaluating the differential abundance of multiple taxa between two groups. In our study, we opted to use the recommended Holm-Bonferroni method [19] and reported only the results with a corrected *P*-value of <0.05. We identified several taxa with different abundance in the two groups; however, most of these results are questionable according to the sensitivity analysis that was performed using the ANCOM-BC2 tool [18] for pseudo-count addition. Like other differential abundance methods, ANCOM-BC2 [18] uses a log transformation on compositional data, such as microbiome data. Nevertheless, zero counts complicate this process, as the logarithm of zero cannot be defined. This issue is commonly addressed by adding a pseudo-count before applying the transformation, but studies have shown that the choice of pseudo-count can influence results, raising the false positive rate [28-30]. ANCOM-BC2 [18] performs a sensitivity analysis to evaluate the impact of pseudo-count addition on its findings, which, in our case, demonstrated that these results are not as stable as it was expected and can be influenced by the choice of different pseudo-counts. This raises concerns that some positive or significant results could be false positives; therefore, we cannot conclusively determine that the taxa that exhibit differences are truly different. 

Although the sensitivity analysis reduced the certainty of the evidence of most results, some differences in the differential abundance analysis remained significant and passed the sensitivity analysis. While Firmicutes and Bacteroidetes showed no overall differences between the two groups, certain Coprococcus species within Firmicutes and specific Bacteroides within Bacteroidota were not evenly distributed. This imbalance may suggest a potential disturbance in the Firmicutes-to-Bacteroidetes (F/B) ratio. More specifically, our analysis showed that several ASVs belonging to the species Coprococcus are in higher abundance in healthy individuals than in patients with AMD, while some Bacteroides ASVs are more abundant in the patients’ group. It has been shown that Coprococcus is one of the short-chain fatty acid (SCFA) producers, particularly generating butyrate [31]. Interestingly, it has been suggested that SCFAs, primarily acetate, propionate, and butyrate, can act as a *double-edged sword*, exerting either inflammatory [32] or anti-inflammatory effects and a neuroprotective effect [33]. Especially butyrate can play a crucial role in maintaining intestinal homeostasis, reinforcing the intestinal epithelial barrier, and exhibiting potent anti-inflammatory effects [34]. Thus, the depletion of butyrate-producing bacteria like Coprococcus could compromise the gut barrier integrity, leading to increased systemic inflammation, which is a risk factor for AMD. On the other hand, Bacteroides are Gram-negative bacteria that possess lipopolysaccharides (LPS) in their outer membranes [35]. Increased abundance of Gram-negative bacteria could lead to the translocation of several proinflammatory LPS into the blood, increasing the risk of inflammation in distal ocular tissues such as the retina or optic nerve [36]. On the grounds of the aforementioned, it can be speculated that both the reduction of SCFAs and the increase in pro-inflammatory taxa such as Bacteroides might contribute to the pathogenesis of AMD via inflammatory pathways.

Similar to our study, other studies have already explored the gut microbiome in patients with AMD using the same techniques as ours. They identified and analyzed 16S rRNA genes and suggested a potential link between intestinal microbiota composition and AMD. A recent study by Zhang et al. [4] showed significant differences between AMD and control groups in gut microbiota and specific gut bacterial taxa. At the phylum level, the AMD group had notably lower proportions of Firmicutes and higher proportions of Proteobacteria and Bacteroidota, resulting in a reduced F/B ratio. On the other hand, these findings were not in line with those of Zinkernagel et al. [9], who observed a shift in the F/B ratio in the opposite direction, with patients with AMD showing a higher relative abundance of Firmicutes and a reduction in Bacteroidetes. Similarly, Zysset-Burri et al. [10] reported an increase in the Firmicutes to Bacteroidetes ratio in patients with AMD, aligning with findings by Zinkernagel et al. [9]. A closer look at the mean relative abundance of Firmicutes and Bacteroidetes reported in these studies reveals considerable variation, while no specific reproducible pattern can be identified (Table 4). 

**Table 4:** Most frequently occurring bacterial phylum in different studies. *Extracted from plots NA, no information available; AMD, age-related macular degeneration; SD, standard deviation

Studies	Mean relative abundance in patients with AMD (SD)		Mean relative abundance in controls (SD)	
	Firmicutes	Bacteroidetes	Firmicutes	Bacteroidetes
Zinkernagel et al. [9]	29.0% (14.4)	65.3% (16.5)	20.4% (8.9)	74.7% (12.6)
Zysset-Burri et al. [10]	42.1% (NA)*	41.6% (NA) *	36.1% (NA)*	40.8% (NA)*
Zhang et al. [4]	65% (NA)*	3.7% (NA)*	79% (NA)*	0.6% (NA)*
Our study	69.7% (9.03)	20.61% (6.70)	70.96% (8.71)	19.11% (7.52)

Besides the contradictory findings about the F/B ratio and relative abundance in the existing literature, there are also conflicting results about the particular genera or species that purportedly differ between the study groups. Each study identified different microbes after performing differential abundance analysis with a specific tool like LEfSe [26], DESeq2 [25], or ANCOM-BC2 [18], etc. In microbiome research, these tools are often used interchangeably in order to identify differentially abundant microbes, but they commonly produce different results [27]. This inconsistency represents a major challenge, not only for the reproducibility and validation of the results across different studies on the same topic but also for drawing accurate biological conclusions from the available data. Two of the abovementioned studies on the gut microbiome of patients with AMD [4,9] have used LEfSe [26], whereas one used MaAsLin [37] for differential analysis. On the other hand, we used ANCOM-BC2 [18] as it is less likely to produce false positive results. It has been suggested that LEfSE [26] should be avoided due to its inappropriate high false discovery rate, while ANCOM-BC2 [18] is a more conservative method [27]. Such differences make comparisons and cross-validation of results challenging, as the analysis by different tools may lead to different results. Despite the different differential abundance tools that were used, our results are in line with other studies [4] that reported higher levels of Bacteroides species in patients with AMD (Table 5).

**Table 5:** Taxonomic features that found to be different between patients with AMD and controls in different studies. AMD, age-related macular degeneration

Studies	Enriched in patients with AMD	Enriched in controls
Zinkernagel et al. [9]	Genus Anaerotruncus, Genus Oscillibacter, Genus Ruminococcus torques, Genus Eubacterium ventriosum	Genus Bacteroides eggerthii
Zysset-Burri et al. [10]	Class Negativicutes	Species of Bacteroides, Genus Oscillibacter
Zhang et al. [4]	Genus Escherichia-Shigella, Genus Bacteroides (up to phylum Bacteroidales)	Genus Blautia, Genus Anaerostipes, Phylum Firmicutes
Our study	Species of Bacteroides	Species of Coprococcus

Another noteworthy limitation of the existing literature [4,9,10] is that the authors of those reports did not mention any differences in the diet and nutritional habits among the participants of their groups. This is important because diet can alter the intestinal microbiome [38]. Besides, there is clear evidence that diet per se constitutes a risk factor for AMD [39-41]. In our study, no participants were vegan or vegetarian or avoided any specific food group, and all adhered to similar food habits. 

A possible limitation of the current investigation is the sample size of 33 patients with AMD and 34 matched controls, which may have affected the power of the study to detect any smaller differences between the two groups. Still, our investigation is one of the most well-powered studies on this topic, as only one other study [10] has actually enrolled more patients.

Considering all these factors, caution is needed to interpret the available results. A few differences were observed in ASV-level between patients with AMD and controls in our sample; however, the absence of sufficient consistency in previous study findings makes it difficult to confidently associate specific gut microbiome patterns with AMD. 

## Conclusions

In this geographically and racially well-defined sample, the intestinal microbiome of newly diagnosed patients with neovascular age-related AMD showed some small but noteworthy differences compared to matched healthy controls. Some Bacteroides ASVs were enriched in AMD patients, while certain Coprococcus ASVs were more abundant in controls. Standardizing methodologies in similar studies and further research on diverse populations are essential to better characterize the potential link between the intestinal microbiome and neovascular AMD.
